# New records of Muscidae (Diptera) from Mediterranean countries

**DOI:** 10.3897/zookeys.496.9445

**Published:** 2015-04-16

**Authors:** Marija Ivković, Adrian C. Pont

**Affiliations:** 1 Department of Zoology, Division of Biology, Faculty of Science, University of Zagreb, Rooseveltov trg 6, 10000 Zagreb, Croatia; 2 Oxford University Museum of Natural History, Parks Road, Oxford OX1 3PW, UK

**Keywords:** *Limnophora*, Croatia, Sierra Nevada, *Coenosia lyneborgi*, Montenegro, Bosnia & Herzegovina

## Abstract

New records are provided for Muscidae from four different Mediterranean countries, with new distribution records for species in ten different genera. Seven species are newly recorded for Croatia, four species for Montenegro and one species for Bosnia & Herzegovina. In this paper we give the first confirmation of an aquatic larval stage for *Lispocephala brachialis* (Rondani, 1877), *Lispocephala spuria* (Zetterstedt, 1838) and *Lispocephala mikii* (Strobl, 1893). A first record of the species *Coenosia lyneborgi* Pont, 1972 since its original description is also provided.

## Introduction

Muscid flies are one of the largest groups of Diptera in Europe, with approximately 600 species (Oosterbroek 2006). The family Muscidae comprises seven subfamilies among which the subfamily Coenosiinae, commonly known as “hunter flies”, is entirely predaceous in both the larval and adult stages. Larvae of the other subfamilies may be predaceous or saprophagous in decaying organic matter, while most adults feed on nectar. Some adults feed on blood or on the tissues of wounded animals (flies of the subfamilies Muscinae and Azeliinae) and they are facultative vectors of diseases (Oosterbroek 2006, Gregor et al. 2002).

In this paper new records of various genera of Muscidae collected from a number of different localities in the eastern Mediterranean and from localities in the Sierra Nevada, Spain, are offered. The fauna of Muscidae from the Mediterranean countries and especially from the eastern Mediterranean is poorly known and has not been sufficiently explored. Apart from the papers by Coe (1960, 1962a,b, 1968a,b) on the countries of the former Yugoslavia, the only recent paper involving the Balkan Peninsula is that by Pont and Ivković (2013) that deals with the genus *Limnophora* Robineau-Desvoidy from some sites in Croatia. “Rearing” records from emergence traps are exclusively from Croatia, while the net-collected records are from different countries, including Croatia. The majority of records are of “hunter flies” as many of them have aquatic larvae and, as all the collecting took place around streams and river banks, the preponderance of these flies was to be expected.

## Material and methods

**Figure 1.**
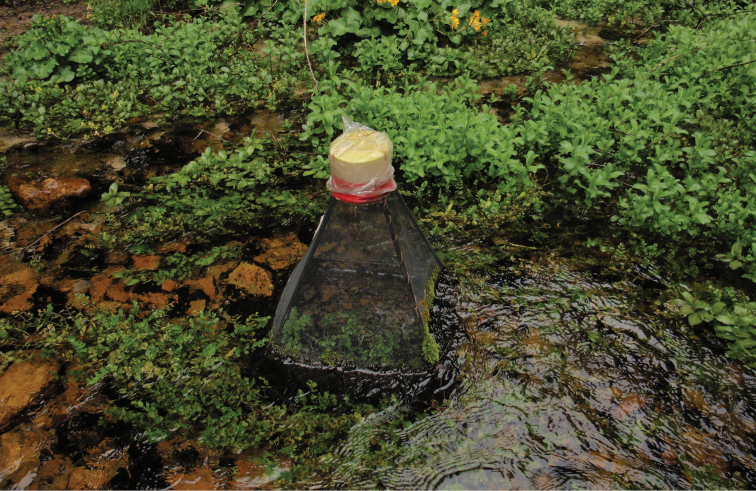
Spring of Bijela Rijeka, Plitvice Lakes, Croatia, emergence trap.

In the course of various ecological and taxonomic projects and surveys by M.I., many muscid flies were collected by means of emergence traps set in streams and small rivers at five sites in Plitvice Lakes National Park and at two sites at Krka National Park, both in Croatia. Traps were emptied once a month, at the end of each month. Each trap had a surface area of 45 × 45 cm (and height 50 cm), was fixed in the sediment of the stream, and contained 2% formaldehyde; six traps were placed at each location (Fig. 1) and for additional details see Ivković et al. (2014). Each trap was recorded with the initial “P” and a number, e.g. “P5" is pyramid emergence trap no. 5. All flies were collected from March 2007 to October 2014. The occasional presence in the emergence traps of a species that does not have aquatic or semi-aquatic larvae is an anomaly that we cannot explain at present. It may be the result of fluctuations in the water level, allowing an adult fly or a drifting puparium to enter the trap. Such anomalies have been discussed by Malicky (2002).

Additional sampling using an aspirator and a sweep net took place between March 2011 and June 2014 at various localities in the eastern Mediterranean part of Europe and from April to June 2013 in the Sierra Nevada, Spain (western Mediterranean). All Muscidae specimens were placed in 80% ethanol and sent to A.C.P. for identification. They were passed through 2-ethoxyethanol (24 hours) and ethyl acetate (24 hours), and then dried, mounted and labelled. Inevitably, many were freshly emerged and not fully hardened, but nevertheless almost every specimen could be identified to species. The monographs by Hennig (1955–1964), Gregor et al. (2002) and Pont and Ivković (2013) were used for identification. All the material listed here is deposited in the Natural History Museum, London, UK (BMNH) and the Oxford University Museum of Natural History, Oxford, UK (OUMNH). GPS coordinates and altitudes for the localities where specimens were trapped and/or collected are given in Table 1.

**Table 1.** The list of sampling sites.

Site name	Longitude	Latitude	Altitude (m)
Río Sucio, Las Barreras (Órgiva), Sierra Nevada, Spain	W 03°26'03"	N 36°54'22"	500
Barranco Frío, Hoya Carlos, Sierra Nevada, Spain	W 03°23'44"	N 36°56'47"	1560
Río Chico, Soportújar, Sierra Nevada, Spain	W 03°24'47"	N 36°55'42"	746
Río Aguas Blancas, Cenes de la Vega, Sierra Nevada, Spain	W 03°30'53"	N 37°09'55"	760
Río Genil, Barranco San Juan, Sierra Nevada, Spain	W 03°23'24"	N 37°08'08"	1200
Río Maitena, Desembocadura, Sierra Nevada, Spain	W 03°24'54"	N 37°09'01"	1018
Djedovica by Rupnica, Papuk Mountain, Croatia	E 17°31'54"	N 45°36'17"	366
Dubočanka stream, Papuk Mountain, Croatia	E 17°40'42"	N 45°29'11"	585
Channel Sava River-Odra River, village Kuče, Croatia	E 16°08'11"	N 45°40'20"	99
*Spring of Bijela rijeka, Plitvice Lakes, Croatia	E 15°33'43"	N 44°50'05"	720
*Upper reach of Bijela rijeka, Plitvice Lakes, Croatia	E 15°33'33"	N 44°50'04"	715
*Upper reach of Crna rijeka, Plitvice Lakes, Croatia	E 15°36'30"	N 44°50'10"	670
*Tufa barrier Labudovac, Plitvice Lakes, Croatia	E 15°35'59"	N 44°52'17"	630
*Tufa barrier Kozjak-Milanovac, Plitvice Lakes, Croatia	E 15°36'32"	N 44°53'39"	545
*Korana village, Plitvice Lakes, Croatia	E 15°37'09"	N 44°55'33"	390
Spring Krčić, Croatia	E 16°19'42"	N 44°01'48"	390
Stream Strmica by Golubić, Croatia	E 16°13'42"	N 44°05'15"	245
Spring of Krka River, Croatia	E 16°14'07"	N 44°02'31"	265
*Roški Slap, Krka River, Croatia	E 15°58'22"	N 43°54'20"	55
*Skradinski Buk, Krka River, Croatia	E 15°57'55"	N 43°48'09"	45
Spring Glavaš, Cetina River, Croatia	E 16°25'48"	N 43°58'36"	385
Jabučica stream, National Park Sutjeska, Bosnia and Herzegovina	E 18°37'02"	N 43°17'24"	767
Spring Bukovica, Durmitor Mountain, Montenegro	E 19°06'42"	N 43°03'30"	1346
Bukovica Stream, Durmitor Mountain, Montenegro	E 19°09'38"	N 43°01'17"	1240
Spring Jeremija, Kolašin, Montenegro	E 19°34'07"	N 42°50'10"	1070
River Murinska rijeka, Montenegro	E 19°53'01"	N 42°39'09"	1000
Alipaša’s springs, Montenegro	E 19°49'33"	N 42°33'00"	930

* use of emergence pyramid traps

## Results

### Faunistic records

The following format is used for the records given here: country, name of the site, followed by the sampling date (in the case of collections from the pyramid emergence traps, the trap number is also given), and the number of sampled specimens. All the sites are listed in Table 1.

### Subfamily Azeliinae

#### Genus Thricops

Taxon classificationAnimaliaDipteraMuscidae

Rondani, 1856

##### Remarks.

A Holarctic genus. The 26 European species are largely confined to higher altitudes. Twelve species are known from the Balkan Peninsula. Adults are well-known to visit flowers, where they feed on both nectar and pollen (Pont 1993). Larvae are terrestrial and are facultative to obligate carnivores (Skidmore 1985).

#### Thricops nigrifrons

Taxon classificationAnimaliaDipteraMuscidae

(Robineau-Desvoidy, 1830)

##### New record.

**CROATIA:** upper reach of Bijela rijeka, Plitvice Lakes, vii.2010, emergence trap P6, 1♀.

##### Comments.

Widespread and common in the West Palaearctic. New for Croatia.

### Subfamily Phaoniinae

#### Genus Helina

Taxon classificationAnimaliaDipteraMuscidae

Robineau-Desvoidy, 1830

##### Remarks.

This a speciose genus, well represented in all biogeographic regions. There are 83 European species, of which18 have been found in the Balkan Peninsula. Adult *Helina* are found in many diverse environments. Larvae are carnivorous and develop mainly in moss or humus soil.

#### Helina moedlingensis

Taxon classificationAnimaliaDipteraMuscidae

(Schnabl, 1911)

##### New record.

**MONTENEGRO:** spring Bukovica, Durmitor Mountain, 5.vii.2012, 1♂.

##### Comments.

Widespread in the Palaearctic, but nowhere common. New for Montenegro.

#### Helina reversio

Taxon classificationAnimaliaDipteraMuscidae

(Harris, 1780)

##### New record.

**CROATIA:** spring Glavaš, Cetina River, 4.vi.2014, 1♀.

##### Comments.

A very common, widespread and eurytopic species in the Palaearctic. Larvae are terrestrial, found in a wide variety of microhabitats (Skidmore 1985).

#### Genus Phaonia

Taxon classificationAnimaliaDipteraMuscidae

Robineau-Desvoidy, 1830

##### Remarks.

Another speciose genus, present in all biogeographic regions. There are 81 European species of which 14 are known from the Balkan Peninsula. Adults are mostly found on flowers or resting on tree trunks, wooden posts, etc. Larvae are carnivorous and live in soil, in fungi and in decaying wood. Some live in the tunnels of wood boring beetles (Scolytidae) and feed on their larvae.

#### Phaonia cincta

Taxon classificationAnimaliaDipteraMuscidae

(Zetterstedt, 1846)

##### New record.

**CROATIA:** tufa barrier Labudovac, Plitvice Lakes, v.2012, emergence trap P6, 1♂.

##### Comments.

Widespread in Europe, but nowhere common. Larvae develop in sap runs in broad-leaved trees where they prey on the larvae of other Diptera. New for Croatia.

#### Phaonia rufiventris

Taxon classificationAnimaliaDipteraMuscidae

(Scopoli, 1763)

##### New record.

**CROATIA:** tufa barrier Kozjak-Milanovac, Plitvice Lakes, ix.2008, emergence trap P5, 1♀.

##### Comments.

A common West Palaearctic species. Larvae have been found in decaying wood and fungi. New for Croatia.

### Subfamily Mydaeinae

#### Genus Hebecnema

Taxon classificationAnimaliaDipteraMuscidae

Schnabl, 1889

##### Remarks.

This is a small genus of some 35 species. There are six species in Europe, five of which are known from the Balkan Peninsula. Larvae are obligate carnivores and live mostly in dung.

#### Hebecnema vespertina

Taxon classificationAnimaliaDipteraMuscidae

(Fallén, 1823)

##### New record.

**CROATIA:** upper reach of Bijela rijeka, Plitvice Lakes, 2.x.2007, emergence trap P6, 1♀.

##### Comments.

A Holarctic species, and common throughout the Palaearctic region. New for Croatia.

### Subfamily Coenosiinae

#### Tribe Limnophorini

##### Genus Limnophora

Taxon classificationAnimaliaDipteraMuscidae

Robineau-Desvoidy, 1830

###### Remarks.

A large genus, found in all biogeographic regions. There are 27 species in Europe of which 13 are known from the Balkan Peninsula. Species of the genus *Limnophora* are usually associated with clean water courses (Rozkošný and Gregor 2004) although Skidmore (1985) writes that *Limnophora riparia* (Fallén) can tolerate high levels of pollution. Both adults and larvae are predaceous (Werner and Pont 2006). The larvae of many species are found among aquatic mosses in streams and rivers (Rozkošný and Gregor 2004). A key to Croatian species, with four new records and one new species, was given by Pont and Ivković (2013).

##### Limnophora caesia

Taxon classificationAnimaliaDipteraMuscidae

(Villeneuve, 1936)

###### New record.

**SPAIN:** Río Chico, Soportújar, Sierra Nevada, 17.iv.2013, 1♀.

###### Comments.

Southern Europe, but an uncommon species.

##### Limnophora croatica

Taxon classificationAnimaliaDipteraMuscidae

Pont & Ivković, 2013

###### New records.

**CROATIA:** stream Strmica by Golubić, 12.iv.2012, 1♀; spring of Krka River, 7.vii.2011, 1♂ 2♀; Roški Slap, Krka River, 6.vii.2011, 1♀; same site, 30.viii.2011, 2♀; same site, 13.x.2011, 3♂ 2♀; same site, 6.xi.2013, emergence trap P1, 3♂ 1♀; same site and date, emergence trap P3, 1♀; same site and date, emergence trap P4, 1♂ 3♀; same site, 5.iii.2014, emergence trap P3, 1♀; same site and date, emergence trap P4, 1♂ 2♀; same site, 2.iv.2014, emergence trap P1, 1♂ 2♀; same site and date, emergence trap P3, 1♂ 2♀; same site and date, emergence trap P4, 6♂ 6♀; same site, 28.iv.2014, emergence trap P1, 1♀; same site and date, emergence trap P3, 1♂; same site and date, emergence trap P4, 4♂ 3♀; same site, 2.vi.2014, emergence trap P1, 1♂ 5♀; same site and date, emergence trap P3, 1♂; same site and date, emergence trap P4, 2♂ 4♀; same site, 26.vi.2014, emergence trap P1, 1♂ 4♀; same site and date, emergence trap P2, 1♂; same site and date, emergence trap P4, 11♂ 18♀; same site, 26.vii.2014, emergence trap P1, 8♂ 5♀; same site and date, emergence trap P2, 1♂ 1♀; same site and date, emergence trap P3, 1♂; same site and date, emergence trap P4, 6♂, 10♀; same site, 2.ix.2014, emergence trap P1, 6♂ 4♀; same site, 2.x.2014, emergence trap P1, 1♀; same site, 27.x.2014, emergence trap P1, 1♀; same site and date, emergence trap P3, 1♂; Skradinski Buk, Krka River, 2.vi.2014, emergence trap P4, 1♂ 1♀; same site and date, emergence trap P6, 1♀; same site, 26.vi.2014, emergence trap P1, 3♀; same site and date, emergence trap P4, 4♂ 7♀; same site and date, emergence trap P5, 1♀; same site, 26.vii.2014, emergence trap P3, 1♀; same site and date, emergence trap P4, 3♀; tufa barrier Labudovac, Plitvice Lakes, viii.2011, emergence trap P5, 1♀; spring of Bijela rijeka, Plitvice Lakes, v.2011, emergence trap P2, 1♂; same site, vii.2013, emergence trap P5, 1♂.

###### Comments.

So far this species is known only from Croatia.

##### Limnophora olympiae

Taxon classificationAnimaliaDipteraMuscidae

Lyneborg, 1965

###### New records.

**CROATIA:** tufa barrier Kozjak-Milanovac, Plitvice Lakes, ix.2009, emergence trap P4, 1♀. **MONTENEGRO:** Alipaša’s springs, 8.vii.2012, 1♂.

###### Comments.

Widespread in the West Palaearctic. New for Montenegro.

##### Limnophora pandellei

Taxon classificationAnimaliaDipteraMuscidae

Séguy, 1923

###### New records.

**SPAIN:** Barranco Frío, Hoya Carlos, Sierra Nevada, 1♀; Río Aguas Blancas, Cenes de la Vega, Sierra Nevada, 13.v.2013, 1♀; Río Maitena, Desembocadura, Sierra Nevada, 13.v.2013, 1♂; Río Genil, Barranco San Juan, Sierra Nevada, 29.v.2013, 1♂ 1♀.

###### Comments.

Widespread in the West Palaearctic.

##### Limnophora pulchriceps

Taxon classificationAnimaliaDipteraMuscidae

(Loew, 1860)

###### New records.

**CROATIA:** Roški Slap, Krka River, 6.vii.2011, 2♂; same site, 13.x.2011, 1♀; same site, 28.iv.2014, emergence trap P1, 1♀; same site and date, emergence trap P4, 2♂ 1♀; same site, 2.vi.2014, emergence trap P2, 1♀; same site and date, emergence trap P4, 1♂ 4♀; same site, 26.vi.2014, emergence trap P1, 1♂ 1♀; same site and date, emergence trap P4, 3♂ 2♀; same site, 26.vii.2014, emergence trap P1, 3♂ 3♀; same site and date, emergence trap P4, 5♂ 2♀; same site, 2.ix.2014, emergence trap P1, 1♂ 2♀; same site, 2.x.2014, emergence trap P4, 1♀; tufa barrier Labudovac, Plitvice Lakes, v.2012, emergence trap P3, 1♂ 1♀; same site, vii.2012, emergence trap P3, 5♂ 16♀; same site, viii.2012, emergence trap P3, 1♂; same site, vii.2013, emergence trap P7, 1♂ 1♀; same site, viii.2013, emergence trap P7, 1♂ 1♀; tufa barrier Kozjak-Milanovac, Plitvice Lakes, ix.2010, emergence trap P5, 1♀; same site, v.2011, emergence trap P4, 1♂; same site, vii.2011, emergence trap P5, 2♂ 4♀; same site, viii.2011, emergence trap P5, 1♀; same site, ix.2011, emergence trap P3, 1♀; Dubočanka stream, Papuk Mountain, 18.ix.2012, 1♂ 1♀.

###### Comments.

Described from Croatia (Dalmatia) and found in southern Europe and the Middle East.

##### Limnophora riparia

Taxon classificationAnimaliaDipteraMuscidae

(Fallén, 1824)

###### New records.

**CROATIA:** Skradinski Buk, Krka River, 6.xi.2013, emergence trap P1, 5♂ 6♀; same site and date, emergence trap P2, 2♂ 1♀; same site and date, emergence trap P3, 3♂; same site and date, emergence trap P5, 1♀; same site and date, emergence trap P6, 1♂ 1♀; same site, 5.iii.2014, emergence trap P1, 2♂ 2♀; same site, 2.iv.2014, emergence trap P1, 2♂ 1♀; same site and date, emergence trap P2, 1♂; same site, 28.iv.2014, emergence trap P1, 1♂ 1♀; same site and date, emergence trap P5, 1♂; same site, 2.vi.2014, emergence trap P1, 1♂ 2♀; same site and date, emergence trap P2, 2♂ 3♀; same site and date, emergence trap P3, 1♀; same site and date, emergence trap P4, 2♀; same site and date, emergence trap P6, 1♀; same site, 26.vi.2014, emergence trap P1, 13♂ 17♀; same site and date, emergence trap P2, 10♂ 4♀; same site and date, emergence trap P4, 9♂ 11♀; same site and date, emergence trap P5, 1♂ 4♀; same site and date, emergence trap P6, 1♂; same site, 26.vii.2014, emergence trap P1, 116♂ 137♀; same site and date, emergence trap P2, 41♂ 95♀; same site and date, emergence trap P3, 12♂ 21♀; same site and date, emergence trap P4, 31♂ 37♀; same site and date, emergence trap P5, 4♂; same site and date, emergence trap P6, 1♀; same site, 2.ix.2014, emergence trap P1, 4♂ 5♀; same site and date, emergence trap P2, 1♂; same site and date, emergence trap P4, 6♂ 10♀; same site and date, emergence trap P5, 1♂ 1♀; same site and date, emergence trap P6, 4♂ 2♀; same site, 2.x.2014, emergence trap P1, 1♀; same site and date, emergence trap P2, 1♀; same site, 27.x.2014, emergence trap P1, 1♀; same site and date, emergence trap P2, 1♂ 1♀; same site and date, emergence trap P4, 1♂, 4♀; Roški Slap, Krka River, 6.vii.2011, 2♂; same site, 6.xi.2013, emergence trap P4, 2♀; same site, 2.iv.2014, emergence trap P4, 1♀; same site, 28.iv.2014, emergence trap P4, 1♀; same site, 26.vi.2014, emergence trap P1, 1♂; tufa barrier Labudovac, Plitvice Lakes, v.2009, emergence trap P2, 1♂; same site, vi.2009, emergence trap P2, 1♂; same site and date, emergence trap P3, 1♂ 1♀; same site, vii.2009, emergence trap P2, 1♂ 2♀; same site and date, emergence trap P3, 1♂ 10♀; same site, viii.2011, emergence trap P2, 1♀; same site and date, emergence trap P5, 2♂ 1♀; same site, ix.2011, emergence trap P1, 1♂; same site, x.2011, emergence trap P2, 1♀; same site, viii.2012, emergence trap P3, 1♀; same site, v.2013, emergence trap P6, 1♂; same site, vi.2013, emergence trap P3, 1♀; same site, vii.2013, emergence trap P2, 1♂; same site, viii.2013, emergence trap P5, 3♀; tufa barrier Kozjak-Milanovac, Plitvice Lakes, vii.2009, emergence trap P5, 1♂; same site, viii.2011, emergence trap P5, 1♀; same site, vi.2012, emergence trap P5, 2♀; same site, vii.2012, emergence trap P5, 1♂ 3♀; same site, ix.2012, emergence trap P5, 1♀; same site, vi.2013, emergence trap P3, 1♂; same site, vi.2013, emergence trap P5, 1♀. **SPAIN:** Río Sucio, Las Barreras (Órgiva), Sierra Nevada, 17.iv.2013, 1♂.

###### Comments.

Widespread and common throughout the Palaearctic region, and closely associated with fast-flowing rivers and streams.

##### Limnophora setinerva

Taxon classificationAnimaliaDipteraMuscidae

Schnabl, 1911

###### New records.

**CROATIA:** spring Glavaš, Cetina River, 3.vi.2014, 1♀; Roški Slap, Krka River, 28.iv.2014, emergence trap P2, 1♂; same site, 2.vi.2014, emergence trap P4, 1♂ 1♀; same site, 26.vi.2014, emergence trap P6, 1♂; upper reach of Bijela rijeka, Plitvice Lakes, viii.2010, emergence trap P3, 1♂; tufa barrier Labudovac, Plitvice Lakes, vii.2013, emergence trap P7, 1♀; tufa barrier Kozjak-Milanovac, Plitvice Lakes, ix.2010, emergence trap P5, 2♂; **MONTENEGRO:** River Murinska rijeka, 11.vii.2013, 1♂; Alipaša’s springs, 11.vii.2013, 2♀; same site, 8.vii.2012, 2♂ 4♀; spring Jeremija, Kolašin, 6.vii.2012, 1♀; Bukovica stream, Durmitor Mountain, 6.vii.2012, 1♂ 1♀. **BOSNIA & HERZEGOVINA:** Jabučica stream, National Park Sutjeska, 4.vii.2012, 1♂. **SPAIN:** Barranco Frío, Hoya Carlos, Sierra Nevada, 17.iv.2013, 1♂ 1♀; Río Chico, Soportújar, Sierra Nevada, 17.iv.2013, 3♂ 1♀; Río Sucio, Las Barreras (Órgiva), Sierra Nevada, 17.iv.2013, 1♂;Rio Genil, Barranco San Juan, Sierra Nevada, 13.v.2013, 1♂; Río Aguas Blancas, Cenes de la Vega, Sierra Nevada, 2♂; same site, 29.v.2013, 2♂; Río Genil, Barranco San Juan, Sierra Nevada, 29.v.2013, 3♂.

###### Comments.

Widespread in the southern Palaearctic and in the Oriental region. New for Bosnia & Herzegovina and Montenegro.

##### Limnophora triangula

Taxon classificationAnimaliaDipteraMuscidae

(Fallén, 1825)

###### New records.

**CROATIA:** tufa barrier Kozjak-Milanovac, Plitvice Lakes, viii.2012, emergence trap P5, 1♀; same site, viii.2012, emergence trap P5, 1♀; Djedovica by Rupnica, Papuk Mountain, 14.vi.2012, 1♀.

###### Comments.

Common throughout the Palaearctic region.

##### Genus Lispe

Taxon classificationAnimaliaDipteraMuscidae

Latreille, 1797

###### Remarks.

*Lispe* is also a large genus, found in all biogeographic regions, with 31 species known from Europe and 14 from the Balkan Peninsula. Adults are predaceous and can be found around standing and running water, where they actively hunt other small invertebrates even in hot, sunny, open habitats (Werner and Pont 2006). Larvae are semi-aquatic and also predaceous, living in organic sand and mud (Skidmore 1985).

##### Lispe tentaculata

Taxon classificationAnimaliaDipteraMuscidae

(De Geer, 1776)

###### New records.

**CROATIA:** Korana village, Plitvice Lakes, 29.vi.2007, emergence trap P1, 1♂ 1♀; same site and trap, 26.vii.2007, 6♂ 1♀; same site and trap, viii. 2008, 2♂ 2♀; same site, 26.vii.2007, emergence trap P2, 1♂; same site, 1.ix.2007, emergence trap P5, 1♀; same site, 29.vi.2007, emergence trap P6, 3♂ 3♀; same site and trap, 26.vii.2007, 7♂ 4♀; same site and trap, viii.2008, 1♂.

###### Comments.

The most widespread species of the genus and common throughout the Palaearctic and Nearctic regions. Adults are aggressive predators of Culicidae and Chironomidae.

##### Genus Spilogona

Taxon classificationAnimaliaDipteraMuscidae

Schnabl, 1911

###### Remarks.

*Spilogona* is a genus primarily of high altitudes and high latitudes. Of the 85 European species, only three are known from the Balkan Peninsula. Adults and larvae are predaceous (Werner and Pont 2006). Adults are mostly found in the vicinity of water, whilst the few known larvae are terrestrial and subaquatic.

##### Spilogona dispar

Taxon classificationAnimaliaDipteraMuscidae

(Fallén, 1823)

###### New record.

**MONTENEGRO:** spring Bukovica, Durmitor Mountain, 5.vii.2012, 1♂.

###### Comments.

Widespread in the western Palaearctic. New for Montenegro.

#### Tribe Coenosiini

##### Genus Coenosia

Taxon classificationAnimaliaDipteraMuscidae

Meigen, 1826

###### Remarks.

A speciose genus, found in all regions. Some 80 species are known from Europe, of which 24 are found in the Balkan Peninsula. Species are found in meadows, forests and damp habitats. Both adults and larvae are predaceous. Larvae are terrestrial, living in a wide range of habitats (Skidmore 1985).

##### Coenosia albicornis

Taxon classificationAnimaliaDipteraMuscidae

Meigen, 1826

###### New record.

**CROATIA:** tufa barrier Labudovac, Plitvice Lakes, v.2009, emergence trap P5, 1♂.

###### Comments.

Widespread in the western Palaearctic. New for Croatia.

##### Coenosia lyneborgi

Taxon classificationAnimaliaDipteraMuscidae

Pont, 1972

###### New record.

**SPAIN:** Río Aguas Blancas, Cenes de la Vega, Sierra Nevada, 13.v.2013, 1♂ 1♀

###### Comments.

This is the first record of the species since its description in 1972, and it is still known only from the Sierra Nevada, Spain. This is a unique species of *Coenosia* as it has only one pair of frontal setae, set high on the frons (see Pont 1972: fig. 1).

##### Coenosia nigridigita

Taxon classificationAnimaliaDipteraMuscidae

Rondani, 1866

###### New record.

**CROATIA:** spring Glavaš, Cetina River, 3.vi.2014, 1♀; channel Sava River-Odra River, village Kuče, 17.iv.2011, 1♀.

###### Comments.

A southern European species. New for Croatia.

##### Coenosia testacea

Taxon classificationAnimaliaDipteraMuscidae

(Robineau-Desvoidy, 1830)

###### New record.

**CROATIA:** upper reach of Crna rijeka, Plitvice Lakes, viii. 2008, emergence trap P4, 1♀.

###### Comments.

Throughout the Palaearctic region.

##### Coenosia tigrina

Taxon classificationAnimaliaDipteraMuscidae

(Fabricius, 1775)

###### New records.

**CROATIA:** Korana village, Plitvice Lakes, 29.vi.2007, emergence trap P4, 1♀; same site, 26.vii.2007, emergence trap P2, 1♀; Channel Sava River-Odra River, village Kuče, 17.iv.2011, 2♂.

###### Comments.

A Holarctic species. The larvae live in the soil and are predators of earthworms (Morris and Cloutier 1987).

##### Genus Lispocephala

Taxon classificationAnimaliaDipteraMuscidae

Pokorny, 1893

###### Remarks.

A small genus in Europe with only 12 species, six of which are known from the Balkan Peninsula. Adults are predaceous on other small insects. No larvae of the European species have been described, but it was suspected that they would be aquatic as the adults are usually found in the vicinity of running water. This is confirmed by the records of the three species given here, all of which were caught in emergence traps set in the water.

##### Lispocephala brachialis

Taxon classificationAnimaliaDipteraMuscidae

(Rondani, 1877)

###### New records.

**CROATIA:** tufa barrier Kozjak-Milanovac, Plitvice Lakes, vi.2012, emergence trap P5, 1♀; spring Krčić, 23.iv.2011, 1♂.

###### Comments.

Central and southern Europe and North Africa. This is the first confirmation of an aquatic life-cycle for this species.

##### Lispocephala mikii

Taxon classificationAnimaliaDipteraMuscidae

(Strobl, 1893)

###### New record.

**CROATIA:** Roški Slap, Krka River, 2.ix.2014, emergence trap P4, 1♂.

###### Comments.

This species was described from Croatia and is a Mediterranean and Afrotropical species. This is the first confirmation of an aquatic life-cycle for this species.

##### Lispocephala spuria

Taxon classificationAnimaliaDipteraMuscidae

(Zetterstedt, 1838)

###### New record.

**CROATIA:** spring of Bijela rijeka, Plitvice Lakes, vii.2012, emergence trap P6, 1♀.

###### Comments.

Throughout the Palaearctic region, but an uncommon species. This is the first confirmation of an aquatic life-cycle for this species. New for Croatia.

##### Genus Spanochaeta

Taxon classificationAnimaliaDipteraMuscidae

Stein, 1919

###### Remarks.

Only two species of *Spanochaeta* are known, *Spanochaeta dorsalis* and an Afrotropical species doubtfully referred to this genus.

##### Spanochaeta dorsalis

Taxon classificationAnimaliaDipteraMuscidae

(von Roser, 1840)

###### New record.

**CROATIA:** Roški Slap, Krka River, 6.xi.2013, emergence trap P4, 1♀.

###### Comments.

Nothing is known of the biology of this species but the present rearing indicates that the larvae are aquatic. Throughout Europe, and also in East Africa. New for Croatia.

## Discussion

The *Fauna Europaea* site for the family Muscidae (Pont 2005) has not been updated since it first went online, and over the past decade a number of new records have been published, new material has been identified by A.C.P., and records in some of the older publications have been re-assessed. For this reason some of the records presented in this paper are not actually new even though they do not appear on the *Fauna Europaea* site. Moreover, the *Fauna Europaea* website did not separate Serbia and Montenegro (Pape et al. 2015).

Including the new records given here, current totals for the countries of the former Yugoslavia are as follows:

Bosnia-Herzegovina: 45 (11 in *Fauna Europaea*)

Croatia: 91 (79 in *Fauna Europaea*)

Slovenia: 93 (85 in *Fauna Europaea*)

Macedonia: 39 (39 in *Fauna Europaea*)

Serbia: 45

Montenegro: 17 (89 in *Fauna Europaea* for Serbia and Montenegro combined)

For comparison, 138 species are known from the Greek Mainland and 258 from Spain. It is evident from these figures that much remains to be discovered about the muscid fauna of the Balkan Peninsula, and areas of mountainous and/or temperate broad-leaf forest should prove to be particularly rich in biodiversity.

## Supplementary Material

XML Treatment for Thricops

XML Treatment for Thricops nigrifrons

XML Treatment for Helina

XML Treatment for Helina moedlingensis

XML Treatment for Helina reversio

XML Treatment for Phaonia

XML Treatment for Phaonia cincta

XML Treatment for Phaonia rufiventris

XML Treatment for Hebecnema

XML Treatment for Hebecnema vespertina

XML Treatment for Limnophora

XML Treatment for Limnophora caesia

XML Treatment for Limnophora croatica

XML Treatment for Limnophora olympiae

XML Treatment for Limnophora pandellei

XML Treatment for Limnophora pulchriceps

XML Treatment for Limnophora riparia

XML Treatment for Limnophora setinerva

XML Treatment for Limnophora triangula

XML Treatment for Lispe

XML Treatment for Lispe tentaculata

XML Treatment for Spilogona

XML Treatment for Spilogona dispar

XML Treatment for Coenosia

XML Treatment for Coenosia albicornis

XML Treatment for Coenosia lyneborgi

XML Treatment for Coenosia nigridigita

XML Treatment for Coenosia testacea

XML Treatment for Coenosia tigrina

XML Treatment for Lispocephala

XML Treatment for Lispocephala brachialis

XML Treatment for Lispocephala mikii

XML Treatment for Lispocephala spuria

XML Treatment for Spanochaeta

XML Treatment for Spanochaeta dorsalis
